# A new family of small ArdA proteins reveals antirestriction activity

**DOI:** 10.1128/jb.00318-25

**Published:** 2025-09-12

**Authors:** A. A. Utkina, A. A. Kudryavtseva, O. E. Melkina, S. M. Rastorguev, A. V. Vlasov, K. S. Pustovoit, I. V. Manukhov

**Affiliations:** 1 Moscow Center for Advanced Studies, Moscow, Russia; 2 Complex of NBICS Technologies, National Research Center “Kurchatov Institute” 68636 , Moscow, Russia; 3 Pirogov Russian National Research Medical University 64882, Moscow, Russia; 4 Joint Institute for Nuclear Research 68634, Dubna, Russia; University of Notre Dame, Notre Dame, Indiana, USA

**Keywords:** ArdA, antirestriction, restriction, modification

## Abstract

Antirestriction proteins protect mobile genetic elements from the host’s restriction-modification (RM) systems. In our study, we identified a new family of small proteins, which we named sArdA. The sArdA proteins are homologous to DNA-mimicking ArdA proteins but differ in size, being approximately one-third the length of full ArdAs. Moreover, the sArdA family contains two subgroups, one of which is structurally similar to the N-terminal end of ArdA, whereas the other one matches the C-terminal end. Both the N-terminal and C-terminal domains of ArdA appear capable of independent expression. Phylogenetic analysis demonstrated that genes encoding these proteins evolved into evolutionarily stable subfamilies, named sArdN and sArdC, respectively. AlphaFold structure prediction of sArdA interaction with RM systems revealed four states of EcoKI, which differ in the angle between its two M-subunits while interacting with different ArdAs or DNA. Interestingly, both sArdN and sArdC triggered the same intermediate closed state of EcoKI, indicating possible new interaction pathways of Ards with RM systems. For phenotypic studies in *Escherichia coli* cells, we cloned the *sardN* gene from the chromosome of *Corynebacterium pilbarense* and the *sardC* gene from *Lactococcus cremoris*. Both genes protected λ phage DNA from restriction by the type I RM system. However, they revealed specificities to different restriction-modification systems. Specifically, *sArdC* was more effective against EcoR124II, whereas *sArdN* was more potent against EcoKI. Furthermore, both genes demonstrated antimethylation activity against EcoKI. Our current findings suggest the idea that the binding specificity of DNA-mimicking proteins to their targets could also be achieved by very short proteins.

**IMPORTANCE:**

Our current findings suggest that the binding specificity of DNA-mimicking proteins to their targets could also be achieved by very short proteins. The ability of these DNA-mimicking proteins to specifically inhibit different DNA-binding proteins makes them a promising tool for regulating a range of intracellular processes, including gene expression.

## INTRODUCTION

Type I RM systems, consisting of specificity subunits, restriction endonucleases, and DNA methyltransferases, provide a critical defense mechanism in bacteria by recognizing and cleaving foreign DNA (1). ArdA proteins can counteract this defense by mimicking the structure and surface charge of DNA, thereby preventing the degradation of a mobile genetic element’s DNA (2). *ard*A genes are commonly found within conjugative plasmids, transposons, and bacteriophage DNA and are typically among the first genes to enter the cell during horizontal gene transfer (3).

Recent evidence shows that chromosomally coded ArdA proteins from *Bifidobacterium bifidum* modulate bacterial gene expression in *E. coli* (4), raising intriguing questions about their cellular roles.

Here, we identify two novel ArdA-like antirestriction proteins from *L. cremoris* (further named ArdA_1576) and *C. pilbarense* (further named ArdA_8247). These newly identified proteins are particularly intriguing due to their exceptionally small sizes, respectively (114 and 88 aa), making them among the smallest ArdA proteins discovered to date.

## MATERIALS AND METHODS

### Bacterial strains and plasmids

*E. coli* strain TG1 (K-12 *gln*V44 *thi-*1 Δ(*lac-proAB*) Δ(*mcrB-hsdSM*)5(*rK–mK–*) F′ [*traD36 proAB + lacIq lacZΔM15*]) was used as the host strain for all experiments. Two novel, exceptionally small chromosomal *ardA* genes were cloned from the *C. pilbarense* B-8247 and *L. cremoris* B-1576 chromosomes. All strains were obtained from the All-Russian Collection of Industrial Microorganisms (VKPM).

### Construction of plasmids

The two novel chromosomal *ardA* genes were amplified from *C. pilbarense* and *L. cremoris* genomes via PCR using primers incorporating NdeI and EcoRI restriction sites. The PCR products were purified and digested with NdeI and EcoRI restriction enzymes. The pIR-DPAl expression vector was also digested with the same restriction enzymes. The digested *ardA* gene fragments and the linearized pIR-DPAl vector were ligated using T4 DNA ligase (Thermo Fisher Scientific, USA). The ligation products were transformed into calcium-competent *E. coli* cells, and transformants were selected on LB agar plates containing kanamycin (50 µg/mL). Plasmids were isolated from positive colonies and confirmed by sequencing. The resulting plasmids (Table 1) contained the target *ardA* genes under the control of the pIR-DPAl expression vector promoter.

**TABLE 1:** Plasmids used in this study

Plasmid	Description	Source
pIR-DPAl	Vector, containing *A. logei* luxR2 gene and LuxR2- regulated promoter	(5)
pIR-DPAl-ArdA-R64	pIR-DPAl vector with *ardA* gene from conjugative plasmid R64 (ArdA_R64 protein)	(6)
pIR-DPAl-ArdA-1576	pIR-DPAl vector with *ardA* gene from the chromosome of *L. cremoris* (ArdA_1576 protein)	This work
pIR-DPAl-ArdA-8247	pIR-DPAl vector with *ardA* gene from chromosome of *C. pilbarense* (ArdA_8247 protein)	This work
pACYCEcoKI	Vector pACYC184, carrying the genes, for IA RMI-system EcoKI	(7)
pKF650	Vector pACYC184, carrying the genes, for IC RMI-system EcoR124II	(8)

### Antirestriction activity assays

To evaluate the antirestriction activity of ArdA_8247 and ArdA_1576 against the EcoKI (GenBank AAC77306.2, AAC77305.1, AAC77304.1) and EcoR124II (GenBank OAF89003.1, CAL6689313.1, OMI69276.1) restriction-modification (RM) systems, unmodified lambda phage (λ₀) was used. Three types of *E. coli* TG1 cultures were used: cells without plasmids, cells harboring plasmids containing the genes for the RM systems, and cells carrying plasmids with the RM system genes along with either *ardA_8247* or *ardA_1576* (as detailed in Table 1). The plasmid pIR-DPAl-ArdA-R64, containing a well-described *ardA* gene from the conjugative plasmid R64, served as a positive control. Phage plating and calculation of the efficiency of plating (EOP) were conducted following established procedures (6).

The efficiency of plating (EOP) was estimated as


  (1) $$EOP_{X}=\frac{N_{X}}{N_{TG1}}$$

where N_X_ is the number of λ_0_ phage plaques on the *Е. coli* TG1 cells carrying genes “X” affecting plaque formation (RMI ± Ard), N_TG1_ is the number of λ₀ phage plaques on *Е. coli* TG1 (without any additional restriction or antirestriction genes).

### Phylogenetic studies

The protein sequences from the NCBI database used to construct phylogenetic trees are presented in Table S1. We used ModelFinder (9) to select the best evolutionary model for our sequence alignments. The phylogenetic relationships between ArdA proteins were visualized using the Tree Of Life (iTOL) v5 online tool (10).

### AlphaFold structure predictions

We performed structure predictions of the proteins ArdA_1576, ArdA_8247, and ArdA_916 as well as their interaction with the EcoKI (S_1_M_2_) protein complex using AlphaFold v.3 software (11). For comparison, the prediction of the interaction between the EcoKI (S_1_M_2_) protein complex and dsDNA (AAAACACGTGTGTGCAA) was performed.

### Antimethylation activity assay

To test the antimethylation activity of newly described sArdA proteins, we used *E. coli* AB1157 [*thr-J leu-6 proA2 his4 thi-J argE3 lacY galK2 ara-14 xyl-5 mtl-l tsx-33 rpsL31 supE44*] strain, which contains the EcoKI RMI system. We collected λ_k_ from the AB1157 strain and λ_k1576_ and λ_k8247_ from AB1157 pIR-DPAl-ArdA-1576 and AB1157 pIR-DPAl-ArdA-8247, respectively. After that, the EOP was estimated.

### Site-directed mutagenesis

To verify our predictions of the EcoKI (S_1_M_2_) protein complex interactions, we tested the effect of point mutations in sArdA proteins on their antirestriction activity against the EcoKI RMI system. We selected D8 and D40 amino acids in ArdA_1576, as well as D7, E75, and E78 in ArdA_8247, and replaced them with uncharged leucines. The oligos used for mutagenesis are listed below:

8247_D7L_F  GAACAACCTG**CT**CAGCACGCCCCGCGTGT

8247_D7L_R  CGTGCTG**AG**CAGGTTGTTCGTGTCCATATG

8247_E7578L_F GATGGGCCCGAAC**TT**GGCTGCT**TT**GTGAGGCCGGAATTCAA

8247_E7578L_R GGCCTCAC**AA**AGCAGCC**AA**GTTCGGGCCCATCTCGCCGCGTACGGGC 

1576_D8L_F GCAGAAAATGATGAA**CT**TTTAGCACAGGAATTAATTGAAC 

1576_D8L_R GTGCTAAA**AG**TTCATCATTTTCTGCTTCCATATGA

1576_D40L_D  GCTTATGGTCGA**CT**TTTAGCGATTGGTGATT

1576_D40L_R  CGCTAAA**AG**TCGACCATAAGCACTAAAGTTAAAAT

We constructed four more pIR-DPAl-based plasmids with mutated sArdA proteins. We also confirmed protein expression using SDS-PAGE (not presented). All mutations were verified using Sanger sequencing.

Antirestriction was measured against the AB1157 strain, which contains the full EcoKI RMI system as described above.

## RESULTS

**Fig 1:**
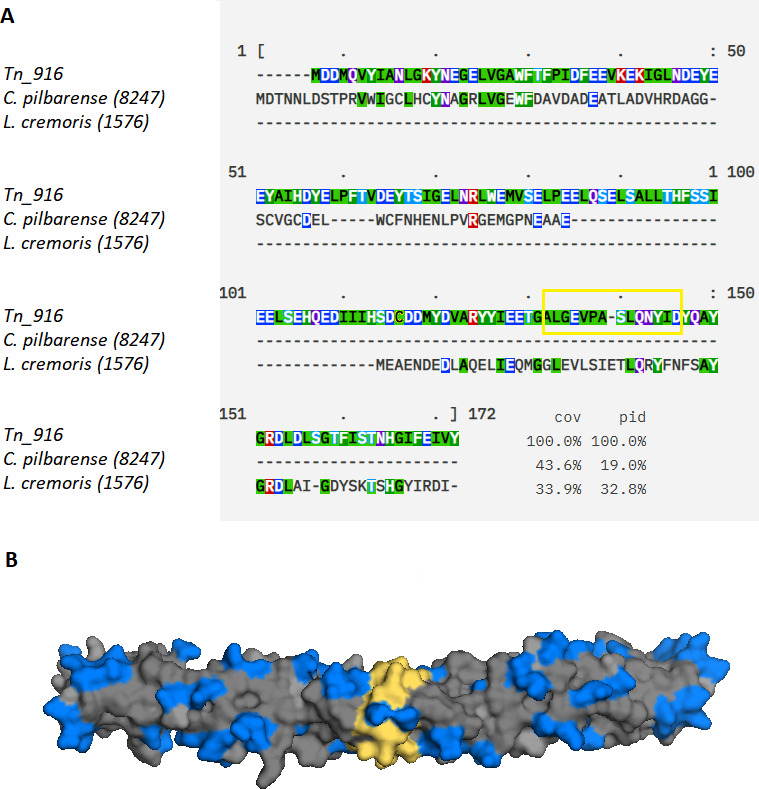
(**A**) Alignment of the amino acid sequences encoded by the *sardA* genes from *C. pilbarense* and *L. lactis* chromosomes. Negatively charged amino acids (D, E) are marked blue. Designations: (–), no homologous residue; cov, coverage; and pid, percent identity. (**B**) The surface of the canonical ArdA protein from the Tn916 transposon (13). Negatively charged amino acids (D, E) are marked blue and form a unique DNA-mimic distribution of negative charge, clearly showing mimicry of the DNA double helix. “Antirestriction motif” or dimerization interface (16, 17) is marked in yellow.

Recently, we showed that *ardA* genes can be found on bacterial chromosomes (4, 12). We searched for *ardA*-like genes across various bacterial species and identified a new group of chromosomal *ardA* genes, which are half the size of classic *ardA* genes such as the one from Tn_916 transposon (13). These small coding sequences are not surrounded by the *ardA* gene fragments but seem to be evolving independently. We named this group of ArdA proteins “small ArdA” (sArdA) and performed a protein alignment using the T-coffee service (14). The alignment results were visualized via the MView web service at the EMBL-EBI site (15) (Fig. 1).

Figure 1 illustrates that although the percentage of amino acid identity is rather low, a distinctive negative charge (indicated by negatively charged amino acids colored in blue), mimicking the DNA structure, is preserved throughout all aligned protein sequences. Subsequently, we generated structural models of the selected proteins, ArdA_1576 and ArdA_8247, using the AlphaFold3 service (11). These modeled structures were subsequently aligned with the canonical ArdA_Tn916 structure (13).

**Fig 2:**
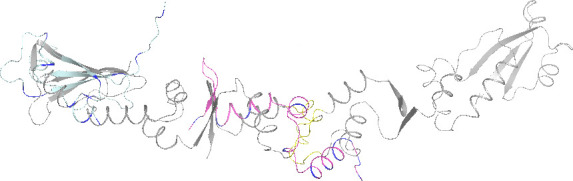
Structural alignment. Gray indicates the dimeric structure of ArdA_Tn916; yellow highlights amino acids forming the interaction interface between two ArdA_Tn916 subunits (‘antirestriction motif’ [16]). Blue indicates the predicted structure of ArdA_8247; pink demonstrates the predicted structure of ArdA_1576. Negatively charged amino acids (D, E) are marked in blue. pIDDT information is presented in Table S2.

From Fig. 2, it is evident that despite the low percentage of identical amino acids (19%), there exists a structural similarity between ArdA_8247 and the N-terminal region of the classical full-length ArdA from Tn916, as well as with the C-terminal segment of ArdA, which aligns to the studied protein ArdA_1576. Moreover, ArdA_1576 aligns not just to the C-terminal region but specifically with the dimerization interface of the full-length ArdA_Tn916, thereby overlapping the dimerization site of its subunits.

**Fig 3:**
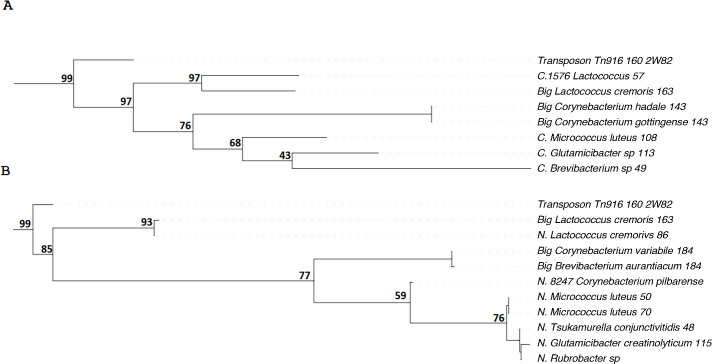
Phylogenetic trees for sArdA proteins from bacterial chromosomes from genus *Lactococcus* and phylum Actinomycetes. The ArdA_Tn916 sequence was used as an outgroup. The “N” before the species name means that the protein aligns to the N-terminus, the “C” means that the protein aligns to the C-terminus, and Big means “full-sized” ArdA. The numbers at the end indicate the protein length in aa. Bootstraps are presented at nodes.

Then, we searched for small *sardA* genes that are homologous to the N- and C-terminal regions of the full-length *ardA* gene across various microorganisms. The results of phylogenetic analysis with the identified representatives of sArdA proteins are presented in Fig. 3. Homologs to the N-terminus are henceforth referred to as sArdN, and those to the C-terminus as sArdC. We also performed a Foldseek (18) structure alignment to confirm N and C division (Fig. S1).

From the data shown in Fig. 3, it is clear that small representatives of the *ardA* gene family are widely distributed among various bacterial species. Specifically, within the phylum *Actinomycetota,* sArdA proteins cluster into two distinct subgroups, or subfamilies. We have provisionally named these subfamilies sArdN (homologs of the N-terminal region) and sArdC (homologs of the C-terminal region of the classical ArdA protein), as depicted in Fig. 3A and B, respectively. The clustering of sArdA across diverse bacterial species shows that once established through evolutionary processes, these genes continue to be preserved, forming clusters of conserved sequences. Hence, here, we demonstrated a convergent evolutionary path of genes *sardN* and *sardC* across at least two bacterial genera.

We note that both sArdN and sArdC from the genus *Lactococcus* cluster with the full-length ArdA protein also present in these bacteria, rather than with their respective subfamilies from other taxa belonging to *Actinomycetes*. This suggests that the small *ardA* genes likely arose multiple times during evolution. Note that sArdN is nearly identical to a large ArdA variant found in *L. cremoris*. This suggests that the protein sArdN likely underwent recent separation or duplication from its ancestral form.

**Fig 4:**
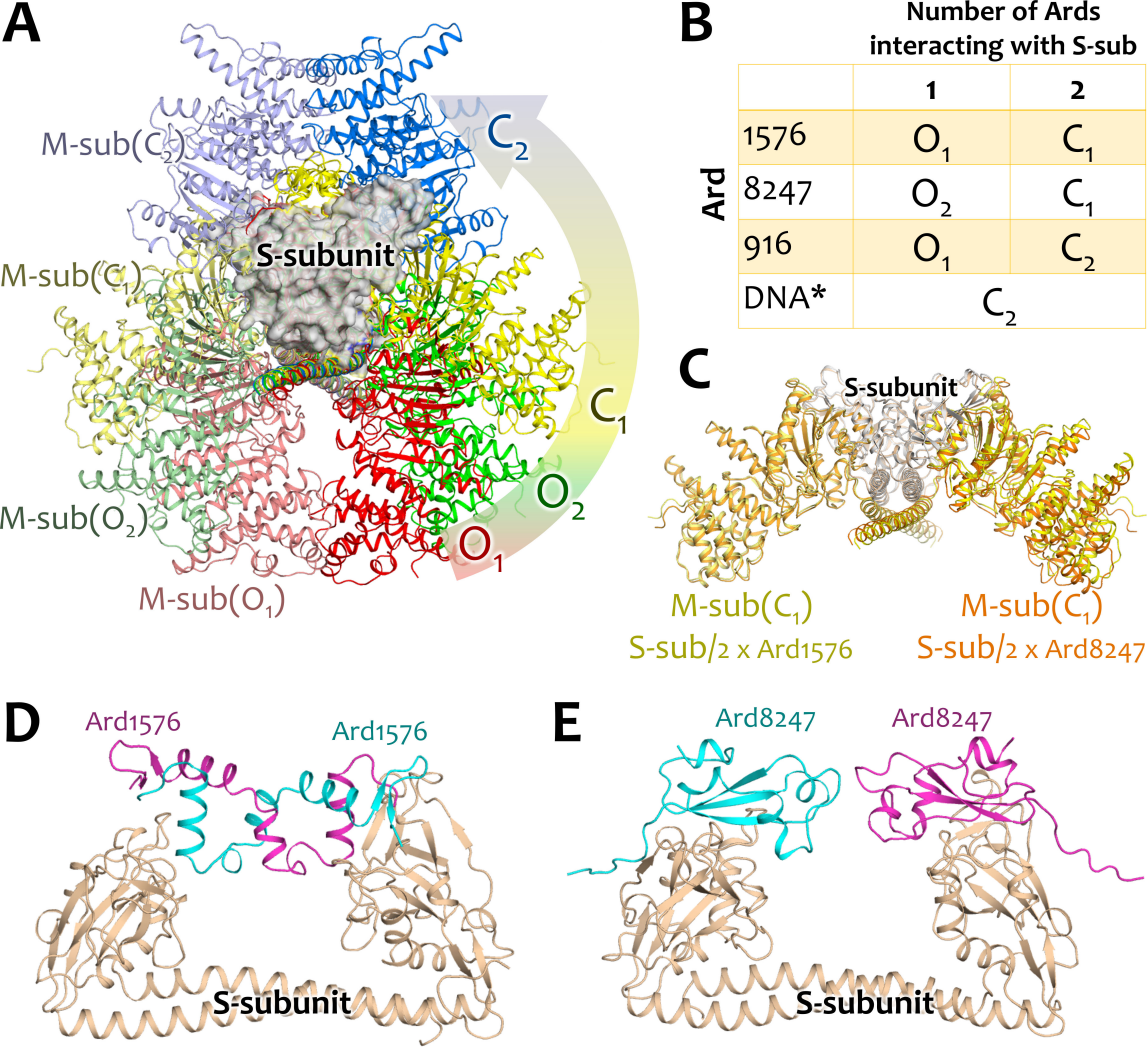
Structure predictions. (**A**) Scheme of the EcoKI protein complex functioning. Two open (O_1_ and O_2_) and two closed (C_1_ and C_2_) states are distinguished. (**B**) EcoKI states during interactions with different agents. *DNA means dsDNA (AAAACACGTGTGTGCAA). (**C**) Structural alignment of EcoKI with 2× sArdC (ArdA_1576) and with 2× sArdN (ArdA_8247). In both cases, the intermediate state C_1_ is predicted. (**D**) Interaction between the S-subunit of EcoKI with 2× sArdC predicts that sArdC forms dimers, and (**E**) with 2× sArdN predicts that two separate sArdN molecules interact with the S-subunit as monomers. pIDDT information is presented in Fig. S3.

To validate our results, we performed structure predictions of EcoKI (S_1_M_2_, one S-subunit, two M subunits) interacting with sArdC and sArdN using Alphafold v.3 (Fig. 4). Interestingly, we revealed four different states of the S_1_M_2_ complex, which feature different angles between M-S-M subunits (Fig. 4A). The intermediate state O_2_ was predicted only in the case of one protein for which sArdN interacts with the S-subunit (Fig. 4B). Interestingly, the state C_1_ was predicted in two cases: 2× sArdC and 2× sArdN interacting with the S-subunit. The structural alignment of the protein complex in the C_1_ state shows highly similar structures and angles between M-S-M subunits (Fig. 4C). A detailed view of the interactions of Ards with the S-subunit reveals that sArdC forms dimers (Fig. 4D), whereas sArdN interacts as two separate monomers (Fig. 4E). This is remarkable because two different agents, sArdC and sArdN, with different interaction pathways nevertheless showed the same conformational changes of the protein complex S_1_M_2_. This fact significantly increases the possibility that the EcoKI-ArdA forms complexes in the predicted C_1_ state in nature. For comparison, other interactions (with DNA and single Ards, *etc*) were also predicted (Fig. S1) and showed that a single sArdC interacts with EcoKI in an open O_1_ state, whereas a single sArdN interacts with EcoKI in an open O_2_ state. Thus, the addition of the second short ArdA might trigger the transition of EcoKI from O_2_ to C_1_ in the case of sArdN and from O_1_ to C_1_ in the case of sArdC, implying that two M-subunits of EcoKI rotate at different angles and skip the O_2_ intermediate open state in the case of EcoKI interacting with a single or two sArdC. The reference predictions with DNA and ArdA_916 triggered the EcoKI closed state C_2_ without any intermediate closed states.

An investigation was performed on the effect of point mutations in sArdA protein models on the state of the EcoKI-ArdA complex (Table S4). For modeling, negatively charged amino acids were selected, which were replaced with uncharged leucines. It turned out that the predicted structural states of S1M2 complexes with sArdC changed the conformation from the C1 state (wild-type sArdC) to state O1 or O2 in several cases. For the sArdN protein, similar amino acid substitutions led to a change in the complex state from C1 (wild-type sArdN) to O1 or O2, and often, there was a complete loss of ability to form a complex with S1M2 EcoKI. Although these are not experimental results (indirect evidence), they may suggest that sArdN forms a less stable complex with S1M2 EcoKI.

**Fig 5:**
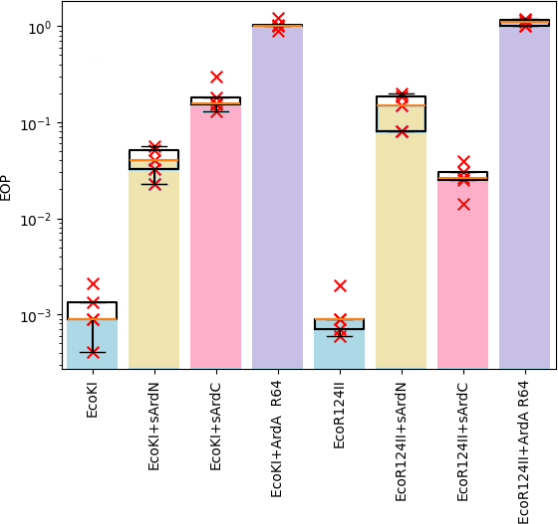
The results of the λ_0_ phage plaquing (EOP) on a lawn of *E. coli* cells containing genes of various RMI systems of gram-negative bacteria. EOP represents a ratio of a phage titer obtained on the experimental lawn relative to the TG1 lawn, which is sensitive to phage infection. Columns: EcoKI, TG1 pACYCEcoKI; EcoKI + sArdN, TG1 pACYCEcoKI, pIR-DPAl-ArdA-8247; EcoKI + sArdC, TG1 pACYCEcoKI, pIR-DPAl-ArdA-1576; EcoKI + ArdA_R64, TG1 pACYCEcoKI, pIR-DPAl-ArdA-R64; EcoR124II, TG1 pKF650; EcoR124II + sArdN, TG1 pKF650, pIR-DPAl-ArdA-8247; EcoAI + sArdC, TG1 pKF650, pIR-DPAl-ArdA-1576; and EcoR124II + ArdA_R64, TG1 pKF650, pIR-DPAl-ArdA-R64. The results of five independent experiments are presented.

AlphaFold predictions allowed us to hypothesize that sArdN and sArdC proteins could work as an antirestriction agent. We confirmed this with lambda phage experiments (Fig. 5). The sArdN and sArdC proteins both exhibit almost «full-sized» antirestriction activity, similar to that of ArdA_R64.

As we can see from Fig. 5, the genes *sardN* and *sardC* have a certain amount of specificity for different RMI systems. Thus, sArdC is more effective against EcoR124II, whereas sArdN is more effective against EcoKI. These findings were confirmed by statistical testing using a one-tailed paired *t*-test (Table S3).

**Fig 6:**
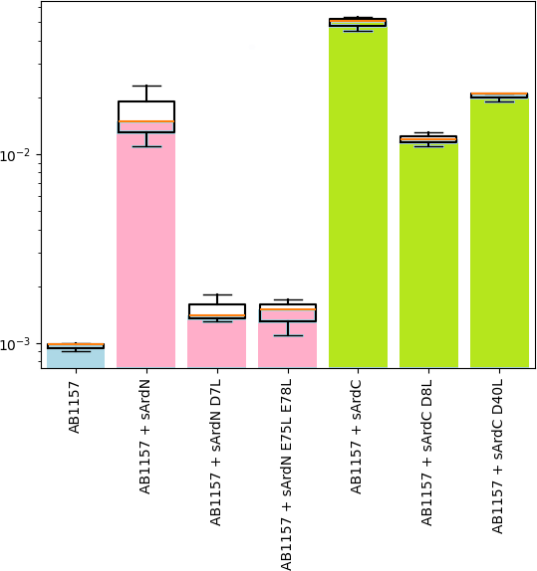
The effect of point mutations on the antirestriction activity of sArdN and sArdC. EOP represents a ratio of a phage titer of phage titers obtained on an experimental lawn relative to the TG1 lawn, which is sensitive to phage infection. The results represent data from three independent experiments.

Then, we chose D8 and D40 amino acids in sArdC, as well as D7, E75, and E78 in sArdN, and replaced them with uncharged leucines. According to our AlphaFold predictions (Table S4), these mutations should lead to an O1 state of the EcoKI complex (or do not form a complex at all), and therefore, the antirestriction effect should decrease against the EcoKI system. Figure 6 shows the results of an EOP test against the lambda phage λ_0_.

Figure 6 demonstrates a decrease in antirestriction activity for both sets of mutations, but it should also be noted that sArdN might be more sensitive to point mutations in negatively charged amino acids (some statistical information is presented in Table S5). Hence, we confirmed more directly that sArdN might form a less stable complex with S1M2 EcoKI.

Additionally, we demonstrated that both studied *sard*-genes inhibit methylation of λ_0_ phage (Table 2). We collected modified λ_k_ phage from three strains: *E. coli* AB1157, which contains the full EcoKI RMI system; *E. coli* AB1157 pIR-DPAl-ArdA-1576; and *E. coli* AB1157 pIR-DPAl-ArdA-8247. Then, we compared the plaquing efficiency of λ_k_ phage from different sources on AB1157 (full EcoKI RMI system) and TG1 (no RMI systems) strains.

**TABLE 2:** Data on the λ phage plating^*b*^

	λ_k_	λ_k_*_sardC_*	λ_k_*_sardN_*	λ_kR64_
The number of phage plaques (−6 dilution)^*a*^ TG1	15,000 ± 200	15,000 ± 400	15,000 ± 450	14,500 ± 500
The number of phage plaques (−6 dilution) AB1157	15,240 ± 400	2,760 ± 70	2,628 ± 120	23 ± 5
Relative protection from restriction	1	1,8 × 10^−1^	1,7 × 10^−1^	1,5 × 10^−3^

^*a*^ The results were averaged over three replicate experiments.

^*b*^ Unmethylated λ_0_ phage or methylated and λ_k_ phages (the latter two were phage λ_0_ after one-step growth in appropriate methylating hosts). All were grown on *E. coli* AB1157 cells containing the complete EcoKI restriction-modification system. ArdA from conjugative plasmid R64 was used as a control.

One of the smallest DNA-mimicking proteins is Ocr from bacteriophage T7, and its DNA mimicry is well-described (19). It is known that DNA mimetic proteins are able to specifically inhibit various DNA-binding proteins. For example, Ugi (from *Bacillus subtilis*) is an inhibitor of the uracil DNA glycosylase (20). Overall, the ability of these DNA-mimicking proteins to specifically inhibit different DNA-binding proteins makes them a promising tool for regulating a range of intracellular processes, including gene expression.

Here, we described two totally new DNA-mimicking proteins that are twice smaller than classic ArdA proteins.

## DISCUSSION

The classical mechanism of new gene acquisition during evolution is considered to be duplication, followed by further mutagenesis of one of the copies (21). In our work, we demonstrated that deletions of different parts of the *ardA* gene lead to the formation of two active gene variants encoding either the C-terminal or N-terminal regions of the full-length protein (Fig. 1 and 2). Phylogenetic studies show that both the C-terminal and N-terminal small ArdA proteins (which we named sArdC and sArdN) are conserved throughout evolution and form homologous genes within certain bacterial taxa (Fig. 3). As seen in Fig. 3A and B, *sardC* genes cluster within taxa such as the *genus Lactococcus* and the phylum *Actinomycetes*. The fact that above the taxon level of *Actinomycetota*, *sArdC* and *sArdN* begin to cluster with their respective full-length ArdA genes strongly suggests repeated formation of small *ardA* genes in bacteria during evolution.

Notably, pairs of sArdA proteins derived from different sources exhibit partial specificity for different restriction-modification systems. For instance, sArdC is more effective against EcoR124II, whereas sArdN is effective against EcoKI. It has been suggested in previous work that the specificity of ArdA proteins to DNA-binding proteins may depend on an additional domain, as observed in *B. bifidum* (4). Structure alignment (Fig. 2) shows that both small antirestriction enzymes are apparently capable of exhibiting the DNA mimicry effect through surface negatively charged amino acids. However, our current findings support the idea that the binding specificity of DNA-mimicking proteins to their targets could also be achieved by very short proteins. This effect opens up prospects for creating agents to specifically inhibit DNA-binding regulators.

AlphaFold structure prediction allowed us to reveal the dynamics of the EcoKI-Ard protein complexes with four different states (two open, O_1_ and O_2_, and two closed, C_1_ and C_2_). Interestingly, that interaction with different agents (2× sArdC and 2× sArdN) provided similar predictions of the EcoKI conformation (C_1_ state). This state, on one hand, is intermediate between open O_1_ and closed C_2_ states (is the angle between M-S-M subunits approximately 120 degrees, Fig. 4A). On the other hand, the EcoKI complex is inhibited during this interaction as shown by the λ_0_ phage plaquing (EOP) on a lawn of *E. coli* cells containing genes of various RMI systems of gram-negative bacteria (Fig. 5); therefore, we inferred that it is in a closed state C_1_. Thus, the possible presence of two distinct closed states of the EcoKI protein complex might indicate totally different molecular mechanisms of inhibiting RM systems, although additional structural studies must be performed to directly test our hypothesis. Notably, amino acid substitution modeling revealed that sArdN is more sensitive to complex formation than sArdC (Table S4). This may be because sArdN does not appear to form a dimer and consequently has fewer protein-protein interactions, leading to less stable binding with S1M2 EcoKI. We also indirectly validated this hypothesis using site-directed mutagenesis: mutations in the negatively charged amino acids of sArdN result in a significantly greater reduction in antirestriction activity.

Structural modeling revealed that sArdC from *L. cremoris* B-1576 is structurally homologous to the interface between subunits of the full-length ArdA dimer. Previous research by Zavilgel’skii and Rastorguev demonstrated that mutagenesis of this interface sequence reduces the anti-restriction activity of ArdA and completely eliminates its antimethylation capability (16, 17). Evidently, sArdC retains the activity of this part of the antirestrictase structure, which appears to have an antimethylase activity. This hypothesis is supported by data presented in Table 2, showing that only about 15% of phage particles grown on r + m + cells containing sArdC are methylated. Surprisingly, sArdN from *C. pilbarense* B-8247, which is homologous to the N-terminus of ArdA and theoretically cannot mimic the subunit interaction interface, is also efficient at antimethylation. This paradox awaits further investigation.

Finally, antimethylation activity combined with relatively weak antirestriction could be beneficial for chromosomal genes during bacteriophage infection or plasmid conjugation.

## SUPPLEMENTAL MATERIAL

Supplemental figures and tables jb.00318-25-s0001.docx Fig. S1 to S5 and Tables S1 to S3.

ASM does not own the copyrights to Supplemental Material that may be linked to, or accessed through, an article. The authors have granted ASM a non-exclusive, world-wide license to publish the Supplemental Material files. Please contact the corresponding author directly for reuse.
