# Supplementary material for: A new family of small ArdA proteins reveals antirestriction activity
Source: J Bacteriol. 2025 Sep 12;207(10):e00318-25. doi: 10.1128/jb.00318-25 (PMC12548430; doi:10.1128/jb.00318-25)
Supplement: Supplemental figures and tables — Fig. S1 to S5 and Tables S1 to S3. [file jb.00318-25-s0001.docx]

**Table S1**. Chromosomal ArdA proteins used for phylogenetic analysis. Big – full sized ArdA antirestriction protein. N – sArdA protein which aligns to a N-terminus of a classic ArdA_Tn916. C – sArdA protein which aligns to a C-terminus of a classic ArdA_Tn916. %QC - query coverage.

| Source | Aligns to | % QC | Size, aa | NCBI number |
| --- | --- | --- | --- | --- |
| Tn_916 | big | 100 | 160 | 2W82 pdb structure |
| *Lactococcus cremoris* | big | 96 | 163 | WP 011835648 1 |
| *Corynebacterium variabile* | big | 87 | 184 | WP 313096454 1 |
| *Glutamicibacter sp* | C | 38 | 113 | WP 283205484 1 |
| *Micrococcus luteus* | C | 48 | 108 | WP 268745131 1 |
| *Brevibacterium sp* | C | 14 | 49 | SMY02182 1 |
| *Corynebacterium hadale* | big | 29 | 143 | CP047655 1 |
| *Tersicoccus phoenicis* | big | 87 | 182 | WP 076705688 1 |
| *Corynebacterium gottingense* | big | 29 | 143 | WJZ13254 1 |
| *Actinobaculum massiliense* | big | 95 | 178 | WP 284907879 1 |
| *Lactococcus cremoris* | N | 41 | 86 | MDA2884301 1 |
| *Brevibacterium aurantiacum* | big | 93 | 184 | MDN6374376 1 |
| *Micrococcus luteus* | N | 18 | 70 | WP NZ JBFOKP010000020 1 |
| *Micrococcus luteus* | N | 18 | 50 | CP043842 1 |
| *Tsukamurella conjunctivitidis* | N | 18 | 48 | WP 146489537 1 |
| *Rubrobacter sp* | N | 36 | 88 | MDN5698578 1 |
| *Glutamicibacter creatinolyticum* | N | 51 | 115 | WP 269780513 1 |
| *Corynebacterium pilbarense* | N | 12 | 78 | MDTNNLDSTPRVWIGCLHCYNAGRL  VGEWFDAVDADEATLADVHRDAGGS  CVGCDELWCFNHENLPVRGEMGPNEAAE* |
| *Lactococcus cremoris* | C | 35 | 57 | MEAENDEDLAQELIEQMGGLEVLSIETL  QRYFNFSAYGRDLAIGDYSKTSHGYIRDI* |

**Table S2.** plDDT for Alphafold structures of sArdA_1576 and sArdA_8247.

| sArdA_1576 (sArdC) | plDDT = 83.92 |
| --- | --- |
| sArdA_8247  (sArdN) | plDDT = 91.88 |


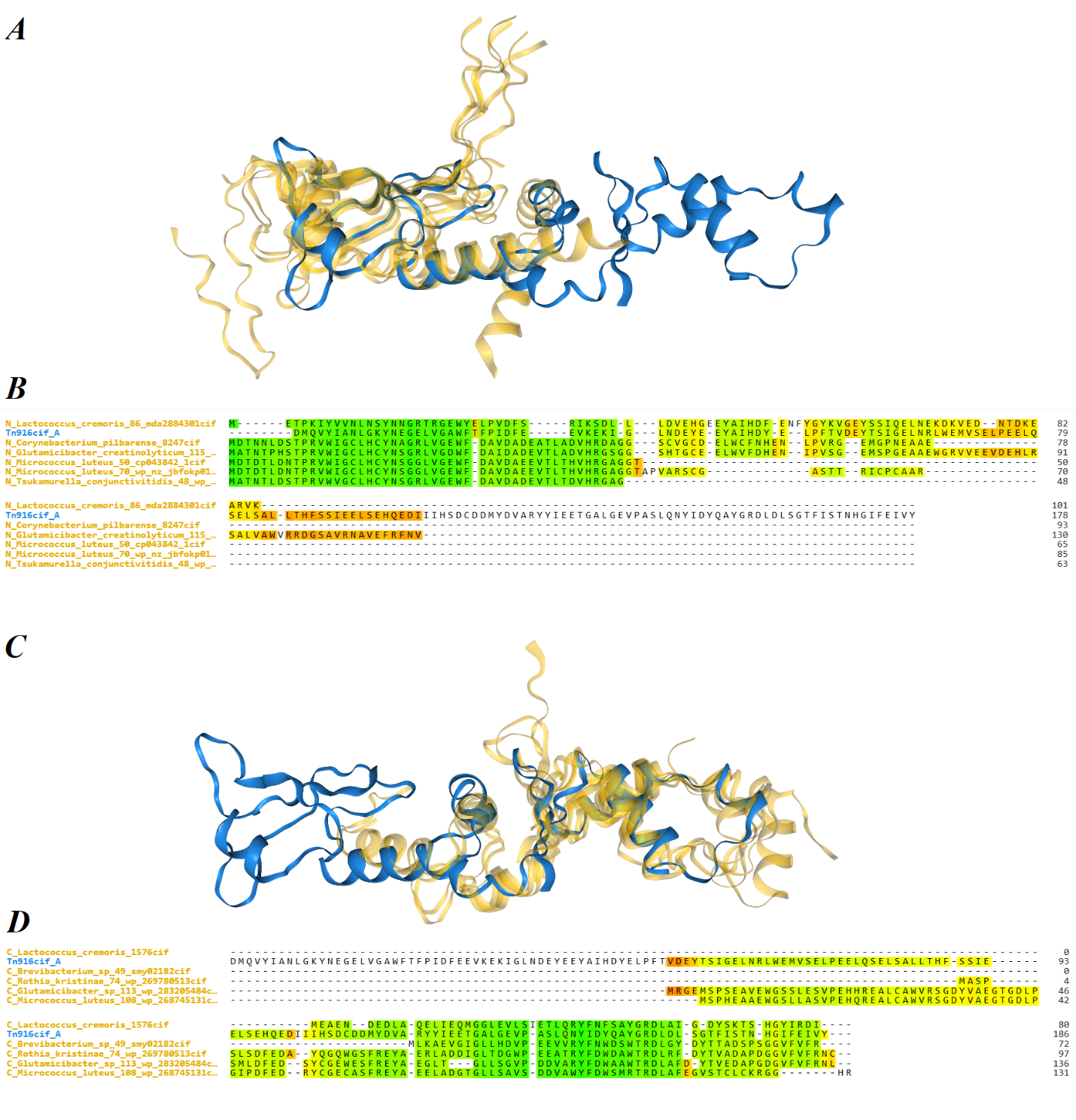
 **Figure S1.** Foldseek structure alignment of the ArdA_Tn916 (blue) protein to sArdN (**A, B**) sArdC (**C, D**) proteins (yellow). **A, C** – structure visualization. **B, D** – amino acids visualization. LDDT values are 0.382 and 0.419 for sArdN and sArdC respectively.


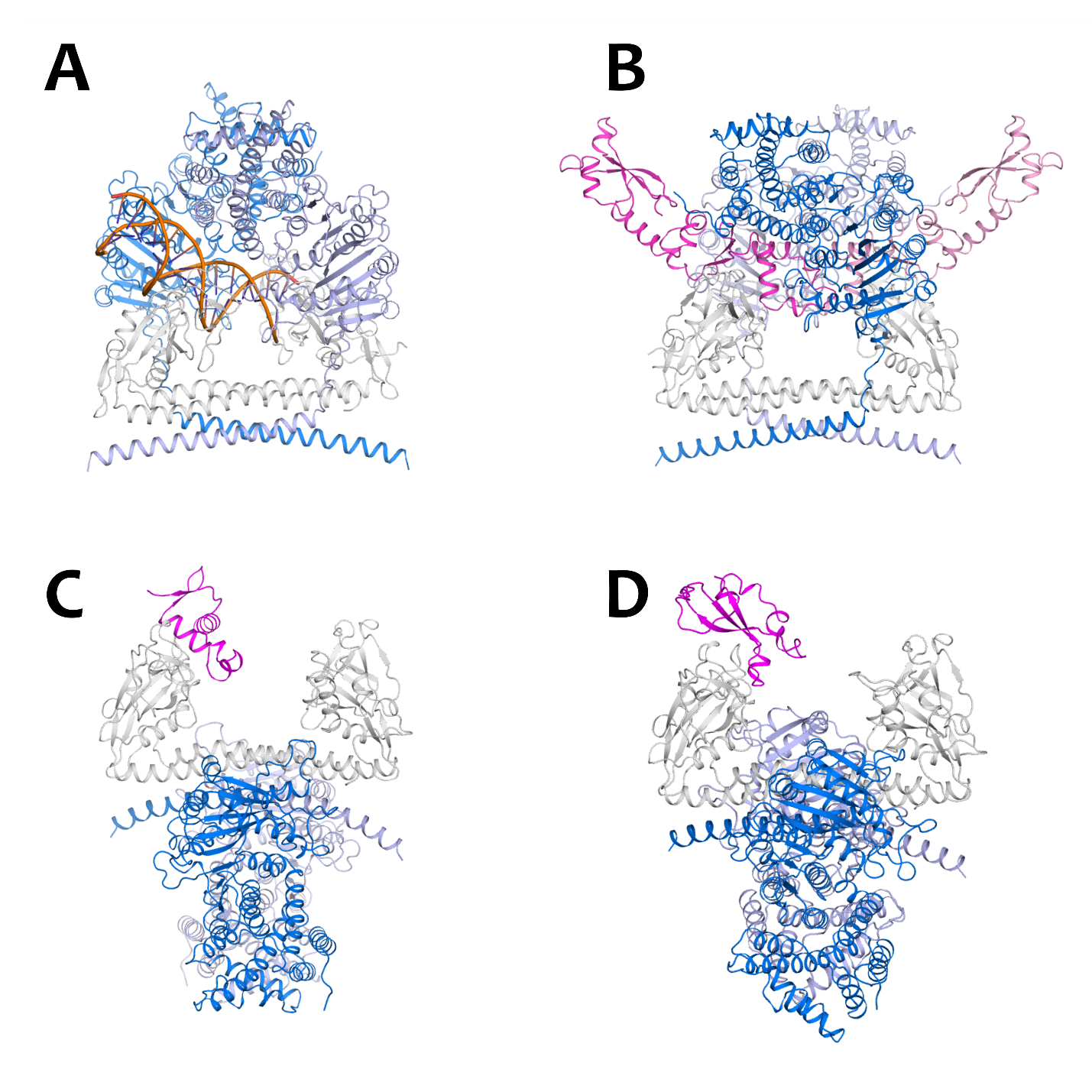


**Figure S2.** Alphafold structure predictions of the EcoKI protein complex with **A** – dsDNA (AAAACACGTGTGTGCAA), **B** - ArdA_916, **C** - ArdA_1576 and **D** - ArdA_8247.

*
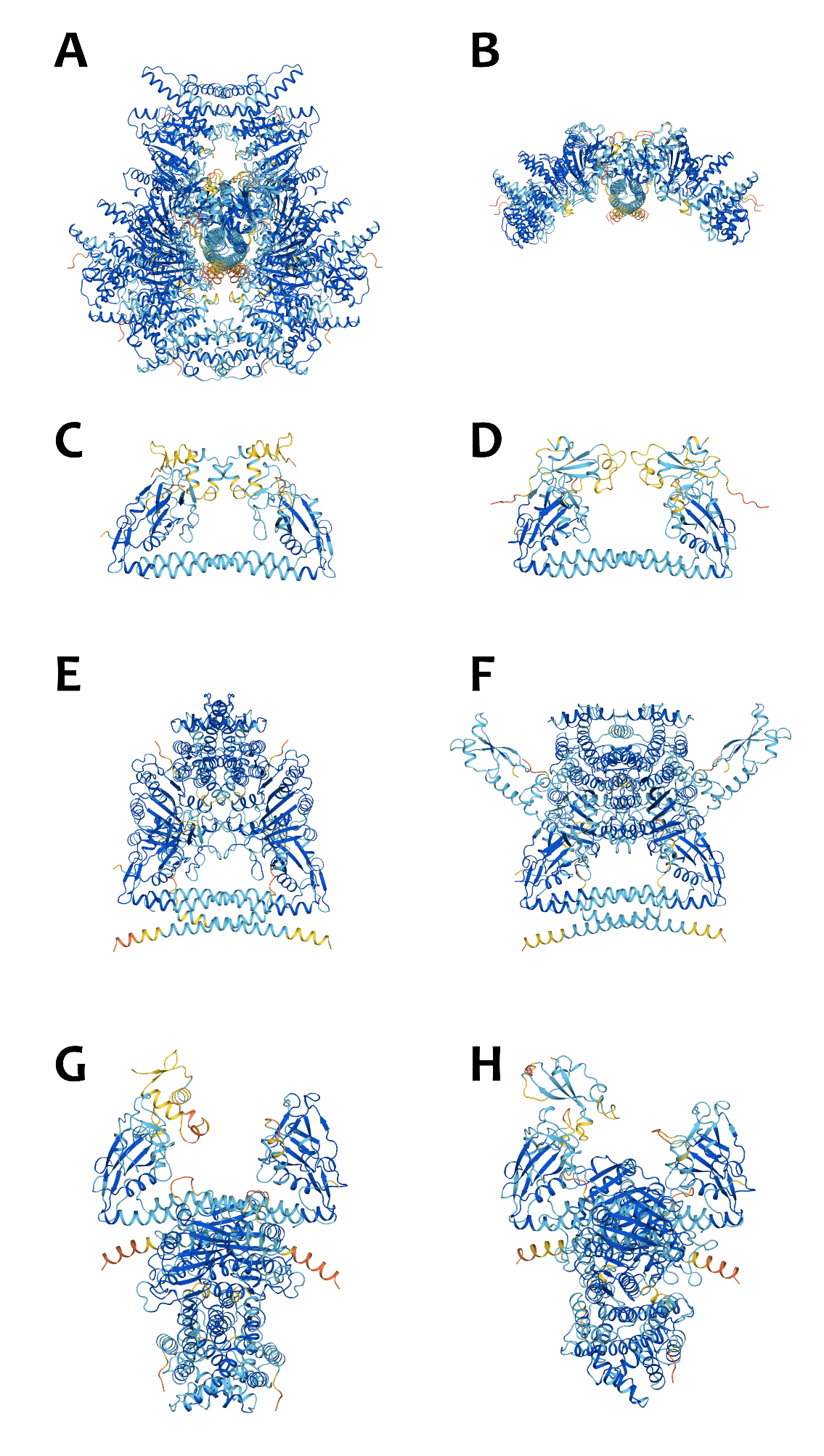
*

**Figure S3.** The confidence of Alphafold-predicted structures. **A -** scheme of EcoKI protein complex functioning. Two open (O_1_ and O_2_) and two closed (C_1_ and C_2_) states are distinguished. **B** – Structural alignment of EcoKI with 2 x sArdC (ArdA_1576) and with 2 x sArdN (ArdA_8247). In both cases the intermediate state C_1_ is predicted. **C** – Interaction between the S-subunit of EcoKI with 2 x ArdA_1576 predicts that sArdC (ArdA_1576) forms dimers, and **D** – with 2 x sArdN (ArdA_8247) predicts that two separate sArdN molecules (ArdA_8247) interact with the S-subunit as monomers. The whole EcoKI protein complex with **E** – dsDNA (AAAACACGTGTGTGCAA), **F** - ArdA_916, **G** - ArdA_1576 and **H** - ArdA_8247. Blue color represents very high confidence (plDDT > 90), light blue represents confident regions (90 > plDDT > 70), yellow represents low confidence (70 > plDDT > 50) and orange – very low confidence (plDDT < 50). PlDDT values for **A** to **H** structures: **A** - O1 (81.41), O2 (81.64), C1 (80.46), C2 (83.91); **B** - 2 x sArdC (ArdA_1576) - 80.46; 2 x sArdC (ArdA_8247) - 80.19; **C** - S with 2 x ArdA_1576 - 78.74; **D** - 2 x sArdN (ArdA_8247) - 78.11; **E** - S2M_dsDNA (AAAACACGTGTGTGCAA) - 85.82; **F** – S with 1 ArdA_916 - 80.42; **G** - S2M with ArdA_1576 - 81.41; **H** - S2M with ArdA_8247 - 81.64.

**Table S3.** Results of the λ.0 phage plaquing (EOP) on a lawn of *E. coli* cells containing genes of various RMI systems of gram-negative bacteria. Statistical analysis using a one-tailed paired t-test demonstrated significant differences in the antirestriction effects between sArdN and sArdC when tested against both EcoKI and EcoR124II.

| Strain | Exp1 | Exp2 | Exp3 | Exp4 | Exp5 | T-test results |
| --- | --- | --- | --- | --- | --- | --- |
| EcoKI+sArdN | 0,0516 | 0,0329 | 0,0229 | 0,04 | 0,056 | The means are significantly different at p < 0.001 |
| EcoKI+sArdC | 0,153 | 0,1832 | 0,3 | 0,13 | 0,1572 |  |
| EcoR124II+sArdN | 0,1844 | 0,2 | 0,15 | 0,0804 | 0,0796 | The means are significantly different at p < 0.001 |
| EcoR124II+sArdC | 0,0264 | 0,039 | 0,025 | 0,03 | 0,014 |  |

**Table S4.** Alphafold predictions of interaction between two Ards with point mutations and S1M2 complex.

| **Protein** | **Mutation** | **S_1_M_2_ complex state** | **pIDDT*** |
| --- | --- | --- | --- |
| **sArdC**  (ArdA_1576) | **WT** | **C_1_** | ipTM = 0.55pTM = 0.64 |
|  | E2L | O_2_ | ipTM = 0.45 pTM = 0.61 |
|  | E4L | O_2_ | ipTM = 0.45 pTM = 0.54 |
|  | D6L | O_2_ | ipTM = 0.53 pTM = 0.63 |
|  | E7L | O_2_ | ipTM = 0.49 pTM = 0.67 |
|  | D8L | O_1_ | ipTM = 0.52 pTM = 0.62 |
|  | E12L | O_2_ | ipTM = 0.47 pTM = 0.54 |
|  | E15L | O_2_ | ipTM = 0.51 pTM = 0.60 |
|  | E21L | C_1_ | ipTM = 0.54 pTM = 0.57 |
|  | E26L | C_1_ | ipTM = 0.52 pTM = 0.62 |
|  | D40L | O_1_ | ipTM = 0.46 pTM = 0.52 |
|  | D56L | C_1_ | ipTM = 0.45 pTM = 0.49 |
| **sArdN**  (ArdA_8247) | **WT** | **C_1_** | ipTM = 0.39 pTM = 0.44 |
|  | D2L | C_1_ | ipTM = 0.41 pTM = 0.40 |
|  | D7L | N/A  Complex doesn’t form | ipTM = 0.35 pTM = 0.44 |
|  | E28L | C_1_ | ipTM = 0.34 pTM = 0.39 |
|  | D31L | C_1_ | ipTM = 0.42 pTM = 0.49 |
|  | D34L | O_2_ | ipTM = 0.42 pTM = 0.44 |
|  | D36L | N/A  Complex doesn’t form | ipTM = 0.37 pTM = 0.45 |
|  | E37L | O_2_ | ipTM = 0.40 pTM = 0.48 |
|  | D46L | C_1_ | ipTM = 0.39 pTM = 0.44 |
|  | D55L | N/A  Complex doesn’t form | ipTM = 0.38 pTM = 0.50 |
|  | E56L | O_1_ | ipTM = 0.42 pTM = 0.47 |
|  | E63L | N/A  Complex doesn’t form | ipTM = 0.39 pTM = 0.47 |
|  | E70L | C_1_ | ipTM = 0.41 pTM = 0.48 |
|  | E75L | O_1_ | ipTM = 0.45 pTM = 0.52 |
|  | E78L | O_1_ | ipTM = 0.38 pTM = 0.46 |

* pIDDT presented as colors below


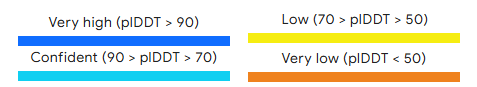


**Table S5.** Results of the λ.0 phage plaquing (EOP) on a lawn of *E. coli* cells AB1157 containing genes of EcoKI RMI system. Statistical analysis using a one-tailed paired t-test demonstrated significant decrease of the antirestriction effects between mutated and WT sArdN and sArdC when tested against EcoKI. AD – antirestriction decrease means the ratio EOP(Ard WT)/EOP(Ard MUT). It is demonstrated a significant difference between the mutations affect on antirestriction activity of sArdN and sArdC.

| Strain | EOP Exp1 | EOP Exp2 | EOP Exp3 | T-test results | AD Exp1 | AD Exp2 | AD Exp3 | T-test results |
| --- | --- | --- | --- | --- | --- | --- | --- | --- |
| AB1157 | 0,001 | 0,0009 | 0,00098 |  |  |  |  |  |
| AB1157 + sArdN | 0,015 | 0,023 | 0,011 |  |  |  |  |  |
| AB1157 + sArdN D7L | 0,0013 | 0,0018 | 0,0014 | The means (comparing to sArdN WT) are significantly different at p < 0.001 | 11,54 | 12,78 | 7,86 | Significantly larger than ADs for sArdC D40L and D8L  p<0.05 |
| AB1157 + sArdN E75L E78L | 0,0017 | 0,0011 | 0,0015 | The means (comparing to sArdN WT) are significantly different at p < 0.001 | 8,82 | 20,91 | 7,33 |  |
| AB1157 + sArdC | 0,045 | 0,053 | 0,051 |  |  |  |  |  |
| AB1157 + sArdC D8L | 0,012 | 0,011 | 0,013 | The means (comparing to sArdC WT) are significantly different at p < 0.001 | 3,75 | 4,82 | 3,92 | Significantly smaller than ADs for sArdN D7L  p<0.05 |
| AB1157 + sArdC D40L | 0,021 | 0,021 | 0,019 | The means (comparing to sArdC WT) are significantly different at p < 0.001 | 2,14 | 2,52 | 2,68 | Significantly smaller than ADs for sArdN D7L  p<0.05 |
